# Sustainable fabrication of dimorphic plant derived ZnO nanoparticles and exploration of their biomedical and environmental potentialities

**DOI:** 10.1038/s41598-024-63459-0

**Published:** 2024-06-12

**Authors:** Bassant Naiel, Manal Fawzy, Alaa El Din Mahmoud, Marwa Waseem A. Halmy

**Affiliations:** 1 Environmental Sciences Department, Faculty of Science, Alexandria University, Alexandria, 21511 Egypt; 2 Green Technology Group, Faculty of Science, Alexandria University, Alexandria, 21511 Egypt; 3 National Egyptian Biotechnology Experts Network, National Egyptian Academy for Scientific Research and Technology, Cairo, Egypt

**Keywords:** Green synthesis, Halophyte, Anti-skin cancer, Antimicrobial, Antioxidants, Mediterranean region, Nanoparticles, Synthesis and processing, Skin cancer, Sustainability, Bacteria, Environmental microbiology, Fungi

## Abstract

Although, different plant species were utilized for the fabrication of polymorphic, hexagonal, spherical, and nanoflower ZnO NPs with various diameters, few studies succeeded in synthesizing small diameter ZnO nanorods from plant extract at ambient temperature. This work sought to pioneer the ZnO NPs fabrication from the aqueous extract of a Mediterranean salt marsh plant species *Limoniastrum monopetalum* (L.) Boiss. and assess the role of temperature in the fabrication process. Various techniques have been used to evaluate the quality and physicochemical characteristics of ZnO NPs. Ultraviolet–visible spectroscopy (UV–VIS) was used as the primary test for formation confirmation. TEM analysis confirmed the formation of two different shapes of ZnO NPs, nano-rods and near hexagonal NPs at varying reaction temperatures. The nano-rods were about 25.3 and 297.9 nm in diameter and in length, respectively while hexagonal NPs were about 29.3 nm. The UV–VIS absorption spectra of the two forms of ZnO NPs produced were 370 and 365 nm for nano-rods and hexagonal NPs, respectively. FT-IR analysis showed Zn–O stretching at 642 cm^−1^ and XRD confirmed the crystalline structure of the produced ZnO NPs. Thermogravimetric analysis; TGA was also used to confirm the thermal stability of ZnO NPs. The anti-tumor activities of the two prepared ZnO NPs forms were investigated by the MTT assay, which revealed an effective dose-dependent cytotoxic effect on A-431 cell lines. Both forms displayed considerable antioxidant potential, particularly the rod-shaped ZnO NPs, with an IC_50_ of 148.43 µg mL^−1^. The rod-shaped ZnO NPs were superior candidates for destroying skin cancer, with IC_50_ of 93.88 ± 1 µg mL^−1^ ZnO NPs. Thus, rod-shaped ZnO NPs are promising, highly biocompatible candidate for biological and biomedical applications. Furthermore, both shapes of phyto-synthesized NPs demonstrated effective antimicrobial activity against various pathogens. The outcomes highlight the potential of phyto-synthesized ZnO NPs as an eco-friendly alternative for water and wastewater disinfection.

## Introduction

Rapid population growth, industrialization, and urbanization are negatively impacting the earth’s aquatic and terrestrial ecosystems by releasing vast quantities of harmful and undesirable substances. Therefore, there are great concerns about the sustainable fabrication of different materials and the mitigation strategies of environmental pollution and issues through Green Chemistry metrics^1^. The fabrication of nanoparticles has received a considerable attention for their unique surface chemistry, morphology, and size that increase their potential in several applications compared to their bulk materials^2^. However, most chemical methods usually pose environmental and health risks through the consumption of hazardous organic solvents, reducing agents, and high energy^3^. Therefore, the need to espouse sustainable, green, and ecofriendly methods to produce nanoparticles has become necessary^4,5^. The phyto-synthesis using various plant extracts is deemed as one of the most valuable green methods because it is easily scalable for industrial use and cost-effective^6^. Numerous phytochemicals, including alcohols, flavonoids, phenols, saponins, terpenoids, and esters, are found in plants. These phytochemicals have the ability to function as mediators that reduce, cap, and stabilize nanoparticles^7,8^.

Among the phyto-synthesized metal oxide nanoparticles, zinc oxide; ZnO has great consideration for its peculiar chemical and thermal stability^9^, biocompatibility^10^, selectivity, and cytotoxic activity against cancerous cells^11^. Accordingly, they have been recently used in numerous biomedical and pharmacological applications, such as antibacterial, antioxidant, anticancer, antifungal, and antidiabetic applications^12–15^. Moreover, studies have reported ZnO NPs as an alternative emergent tool successfully utilized for water disinfection against numerous microbial strains such as *Aspergillus flavus, Escherichia coli, Candida albicans,* and *Staphylococcus aureus*^16,17^. Poor or low water quality causes the emergence of > 50 kinds of diseases that causes 80% of illnesses^18^. It has been reported that ~ 829 thousands of people die from diarrhea spurred by unclean water and sanitation. Contaminated water resulting from inappropriate management of industrial, agricultural, and domestic wastewater is the vehicle for transmission of water and food-borne pathogens. Furthermore, the consumption of polluted, unclean water in food processing and irrigation influences the spread of food-borne diseases and the loss of crops^19,20^. These pathogens cause severe skin, gastrointestinal and respiratory infections^18^. In this regard, ZnO nanoparticles could be utilized as a good antimicrobial agent without harmful byproducts for the protection of environmental and public health.

The production of nanoparticles with defined sizes and shapes remains difficult^2^. Several factors were previously reported to affect the resultant nanoparticle characteristics, such as reaction temperature, drying temperature, reaction time, pH, precursor, and extract concentration^21–24^.

Different plant species have been reported for the fabrication of polymorphic, hexagonal, spherical, and nano-flower ZnO NPs with various diameters^25–28^. However, a few studies succeeded in synthesizing small diameter ZnO nanorods from plant extract at ambient temperature^22,29^.

In the current research, we explored the potential of utilizing the aqueous extract of a common Mediterranean salt marsh plant, *Limoniastrum monopetalum* (L.) Boiss as reducing and capping agent for the ZnO NPs fabrication for the first time. Salt marsh vegetation is an essential class of vegetation especially in the Mediterranean region with ecologic and economic value. The selected species is a member of the family Plumbaginaceae^30^. It is a traditional medicinal plant famous for its antidysenteric activity and also exhibits antioxidant and antibacterial effects, which can be linked to the substantial amount of phenolic chemicals it contains, notably vanillic and gallic acids^31,32^. Accordingly, the specific objectives of the present work were to: (1) elucidate the reaction temperature that affects the form and size of the produced ZnO NPs. (2) Evaluate the antimicrobial, cytotoxicity and antioxidant capability of the phyto-prepared ZnO NPs and (3) Evaluate the impact of shape and size variation of ZnO NPs on their potentialities in biomedical and environmental applications.

The successful synthesis of small-diameter ZnO NPs with diverse shapes using the aqueous extract of *L. monopetalum* at ambient temperature represents a pioneering approach. This attempt opens avenues for exploring additional Mediterranean salt marsh plant species for green NPs fabrication, promising further innovation and advancements in the field of phytosynthesis of NPs.

## Material and methods

### Plant material collection and processing

Field surveys were conducted along the salt marshes habitat in El-Alamein region situated at the western Mediterranean coastline of Egypt (Latitude 30° 55′ 338″, Longitude 28° 29′ 365″, Altitude 11) for the collection of *L. monopetalum* specimens. The relevant national and international guidelines^33^ were followed during the plant material collection. The permission for the collection of the species for the study purposes was acquired from the Department of Environmental Sciences at Alexandria University. The identification of the collected plant specimens was conducted by Dr. Marwa Waseem A. Halmy following Täckholm^34^ and Boulos^35^. Voucher specimens were prepared and placed in the public herbarium of Tanta University (TANE) under voucher numbers 14210–14220. The plant grinded aboveground parts were prepared as described in our previous work^36^. Briefly, the plant aerial parts were thoroughly washed with running tap water and then deionized water. Eventually, it was dried at 60 °C, ground to a fine powder, and stored in sealed containers.

#### Sustainable ZnO NPs fabrication from *L. monopetalum* extract

20 g L^−1^ of the *L. monopetalum* powder was added in distilled water (DW) at 70 °C for 30 min, left to cool at ambient temperature, then filtered using filter paper (Whatman), and then stored at 4 °C for further use.

For the phyto-synthesis of ZnO NPs; a simple, low cost, and energy efficient approach was implemented as stated by Naiel et al.^36^. *L. monopetalum* extract was mixed with 0.5M Zinc acetate dihydrate [Zn (CH_3_COO)_2_⋅2H_2_O] in a ratio of (1:10). Subsequently, 2 M sodium hydroxide [NaOH] was added drop by drop to maintain the mixture pH to 12, the reaction was maintained at ambient temperature.

**Figure 1:**
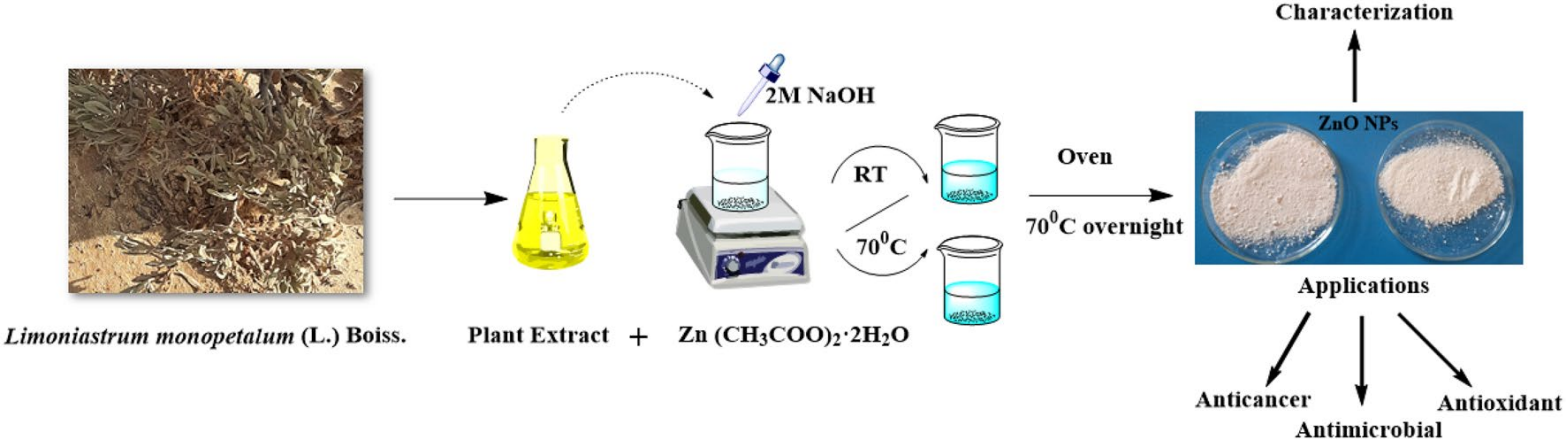
Phytosynthesis of ZnO NPs using *L. monopetalum* aqueous extract.

The effect of the reaction temperature on the synthesis of the ZnO NPs was assessed by preparing another mixture. This mixture was prepared and stirred at 70 °C for 30 min until a color change from yellow to white was observed and started to precipitate indicating the completion of the reduction process and formation of ZnO NPs^36–39^. The formed precipitate was then decanted, washed with DW, and dried-up at 70 °C overnight to produce ZnO NPs powder with two different shapes. The produced powder was kept at ambient temperature in sealed bottles for additional characterization and application. The process is illustrated in Fig. 1.

### ZnO NPs physicochemical characteristics

The characterization of phyto-synthesized ZnO NPs produced from *L. monopetalum* aqueous extract was conducted by several methods to determine their physicochemical characteristics as listed in the Supplementary Information (S1).

### Anticancer activity and cytotoxicity

The cytotoxic and /or cell viability activity of the two produced ZnO NPs were examined in vitro by the colorimetric and quantitative MTT (Methylthiazolyl diphenyl-tetrazolium bromide) assay, and Epidermoid Carcinoma (A-431) cell lines. For the cytotoxicity and biocompatibility evaluation, the formed ZnO NPs were tested against normal fibroblasts (HFB4) at various concentrations. The commonly used type of skin cancer, Epidermoid Carcinoma (A-431) cell lines were chosen for the study^40,41^. Cell lines were obtained from the Vacsera Center in Egypt. The ZnO NPs (the range of 31.25–1000 μg mL^−1^) were examined against the A-431 and HFB4 cell lines. A detailed methodology is reported in the Supplementary Information (S2).

### Antioxidant activity

The 2,2-diphenyl-1-picrylhydrazyl; DPPH assay was implemented to evaluate the antioxidant capability of the phyto-synthesized ZnO NPs. To summarize, 0.1 Mm of 1 mL DPPH was mixed with 3 mL of the dissolved ZnO NPs in ethanol at 1.95–1000 µg mL^−1^ concentrations. The mixture was maintained at pH range of 5–6.5. After that, stirring vigorously and kept at ambient temperature for 30 min. The method was done in triplicate and the absorbance was estimated at 517 nm using UV–VIS spectrophotometer (Milton Roy, USA). DPPH Scavenging Activity (%) was calculated and expressed as IC_50_^42,43^:

1 $$\frac{{A}_{0}-{A}_{1}}{{A}_{0}}\times 100$$

where A_0_ is the control absorbance, and A_1_ is the test absorbance.

### Antimicrobial activity

The effect of the plant-mediated ZnO NPs using *L. monopetalum* as potential antimicrobial agents was investigated against pathogenic species. The study focused on pathogenic microorganisms responsible for medical and environmental implications, particularly:

i) Gram-positive bacteria [*Bacillus Subtilis* (ATCC 6633), *Staphylococcus aureus* (ATCC 6538)].
ii) Gram-negative bacteria [*Pseudomonas aeruginosa* (ATCC 90274), *Escherichia coli* (ATCC 8739)],
iii) Pathogenic fungi [*Aspergillus flavus* and *Candida albicans* (ATCC 10221)].

Gentamycin was utilized as a positive control for bacterial strains, whereas Fluconazole was used as control for fungal species. A total of 100 µl of phyto-synthesized ZnO NPs and positive controls were used in the assessment. Antimicrobial activity was detected via the estimation of the inhibition zone diameter (in mm) by means of the ‘agar well diffusion’ method^44–46^.

## Results and discussion

### Physicochemical descriptions of ZnO NPs

#### Surface properties and phytochemical profile

**Figure 2:**
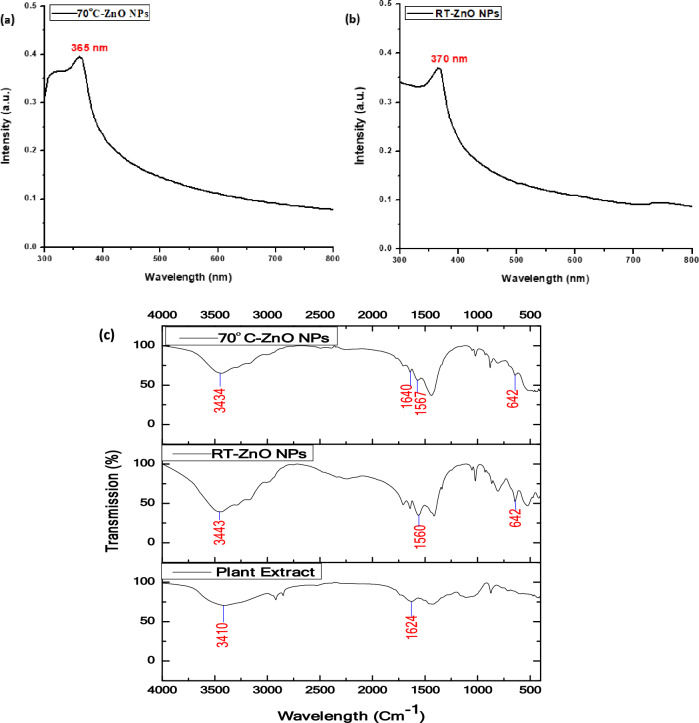
(**a**, **b**) UV–Vis spectra of the phyto-synthesized ZnO NPs and (**c**) FT-IR spectra of *L. monopetalum* extract and the phyto-synthesized ZnO NPs.

The spectra of the two ZnO NPs forms attained different intensity peaks; the form prepared at ambient temperature appeared at 370 nm, while that prepared by heating to 70 °C emerged at 365 nm (Fig. 2a,b). The two peaks are characteristic and distinct for ZnO NPs formation. These results are consistent with earlier studies^7,47^.

The functional groups of FT-IR revealed the detection of Zn–O stretching at 642 cm^−1^ indicating the successful synthesis of ZnO NPs as reported by Khalaf et al.^23^. The plant extract spectrum had a broad peak at 3410 cm^−1^ corresponding to–OH group. This peak was shifted in the ZnO NPs FT-IR spectrum of both ZnO NPs to 3443 cm^−1^ and 3434 cm^−1^ for rod-shaped and hexagonal NPs, respectively, similar to Rani et al.^48^. These peaks may be correlated with the phenolic, alcoholic, and terpenoid content of the plant extract. As depicted in Fig.2c, 1624 cm^−1^ peak in the plant extract spectrum corresponded to H–O–H bending, which shifted to 1560 cm^−1^, 1567 cm^−1^, and 1640 cm^−1^ in the spectrum of ZnO NPs. The peaks of plant extract shifted to another wavelength or disappeared in the FT-IR spectrum of the gained ZnO NPs, demonstrating the importance of these phytochemicals for the stability and reduction of ZnO NPs^49^.

**Figure 3:**
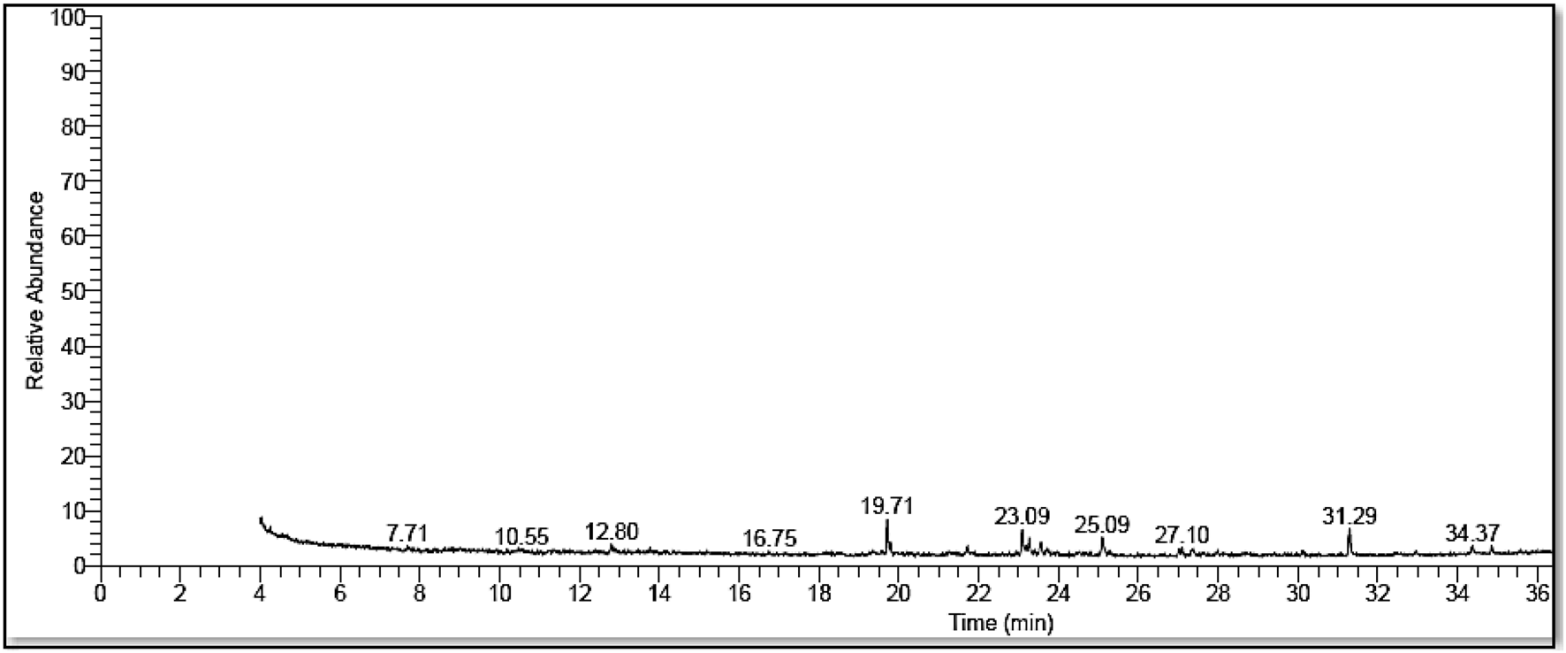
GC spectrum of L. monopetalum extract.

The analysis of the phytochemical profile of the plant extract using Gas Chromatography–Mass Spectrophotometry (GC–MS) helped identify the phytochemicals that might be accountable for reducing ZnO NPs and stabilizing its formation (Fig. 3 and Table 1). Four categories of bioactive compounds were identified and classified into monoterpenoid alcohols, oxanes, and esters. The main compounds were identified in Table 1. These phytochemicals may have acted as reducing, stabilizing, and capping agents for ZnO NPs, as suggested by previous studies^50–52^. These phytochemicals contain hydroxyl groups that could assist in the reduction of the precursor zinc acetate dihydrate to ZnO NPs. They are also responsible for the development of the stable form of ZnO NPs via capping process^53^. Moreover, the aforementioned FT-IR analysis was consistent with the occurrence of the bioactive compounds detected by the GC–MS.

**Table 1:** Phytochemical compounds identified in the extract of *L. monopetalum.*

No.	Compounds	Chemical structure	Category	Retention time (min)	Area (%)
1	Cis-p-Mentha-2,8-dien-1-ol	C_10_H_16_O	Monoterpene alcohol	19.71	13.94
2	Cis-5,8,11,14,17-Eicosapentaenoic acid	C_20_H_30_O_2_	Fatty acid methyl ester	23.09	11.48
3	Bisabolol oxide A	C_15_H_26_O_2_	Oxanes	25.10	11.2
4	Hexadecanoic acid, TMS	C_19_H_40_O_2_Si	Ester	31.29	12.19

#### Morphological structure

**Figure 4:**
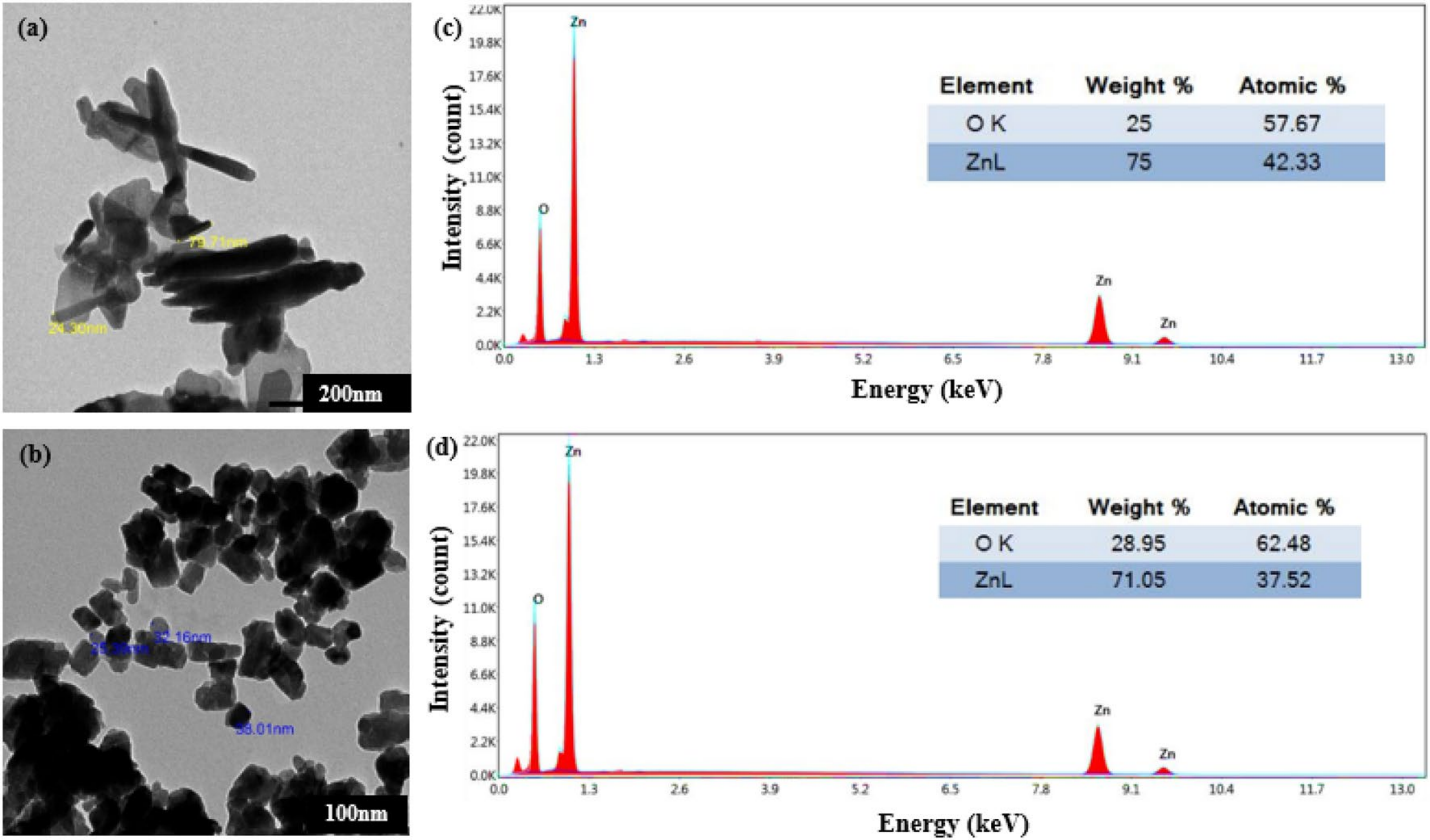
TEM images of the phyto-synthesized ZnO NPs, (**a**) RT, (**b**) 70 °C and EDX spectrum (**c**) and (**d**).

The data acquired from Transmission Electron Microscope (TEM) and Energy Dispersive X-ray (EDX) revealed that two different morphologies of ZnO NPs were obtained with varying reaction temperatures (see TEM images Fig. 4). Rod-shaped ZnO NPs were formed at ambient temperature, and the nearly hexagonal shape was formed at 70 °C. The average size of rod-shaped ZnO NPs was ~ 25.3 nm and 297.9 nm in diameter and in length, respectively whereas that of the hexagonal NPs was ~ 29.3 nm as demonstrated in Fig. 4a,b respectively. These results followed with Kotresh et al.^54^ who revealed that reaction temperature is a key factor affecting the morphological features of nanoparticles. In contrast to our results; Thi et al.^22^ reported large coagulated ZnO nano-rods with lengths and widths of ∼ 370 nm and ∼ 160 nm, respectively using orange peel extract. A summary Table 2. Illustrating the previously prepared ZnO NPs using various reaction temperatures and plant extracts.

**Table 2:** Comparison between previously prepared ZnO NPs in literature using various plant extracts and reaction temperatures.

Phyto-synthesized ZnO NPs	Reaction temperature (°C)	Shape	Size (nm)	References
*Leea asiatica* leaf extract	RT	Nanorods	218 nm	^29^
Orange peel extract	60 °C	Nanorods	160 nm-diameter 370 nm-length	^22^
*Origanum majorana* leaf extract	40 °C	Nanorods	90 nm–125 nm-diameter 0.5 μm–1.2 μm-length	^55^
*Avena Sativa L seed extract*	80 °C	Hexagonal	100 nm	^56^
*Prosopis farcta (Banks & Sol.) J.F. Macbr*	60 °C	Hexagonal	40–50 nm	^57^
*L. monopetalum* aerial parts extract	RT	Nanorods	25.3-diameter 297.9 length	Current study
*L. monopetalum* aerial parts extract	70 °C	Hexagonal	29.3 nm	Current study

The EDX spectra affirmed the occurrence of two robust peaks with high intensities of Zinc and Oxygen at ~ 1keV and ~ 0.5keV respectively (Fig. 4c,d). The atomic percentage of Zn was 42.33% and Oxygen was 57.67% for rod-shaped ZnO NPs. On the other hand, the atomic percentage of Zn was 37.52% and oxygen was 62.48% for the near-hexagonal NPs (Fig. 4c,d). No impurities were detected, confirming the purity of the phyto-synthesized NPs^58^.

#### X-ray diffraction (XRD)

**Figure 5:**
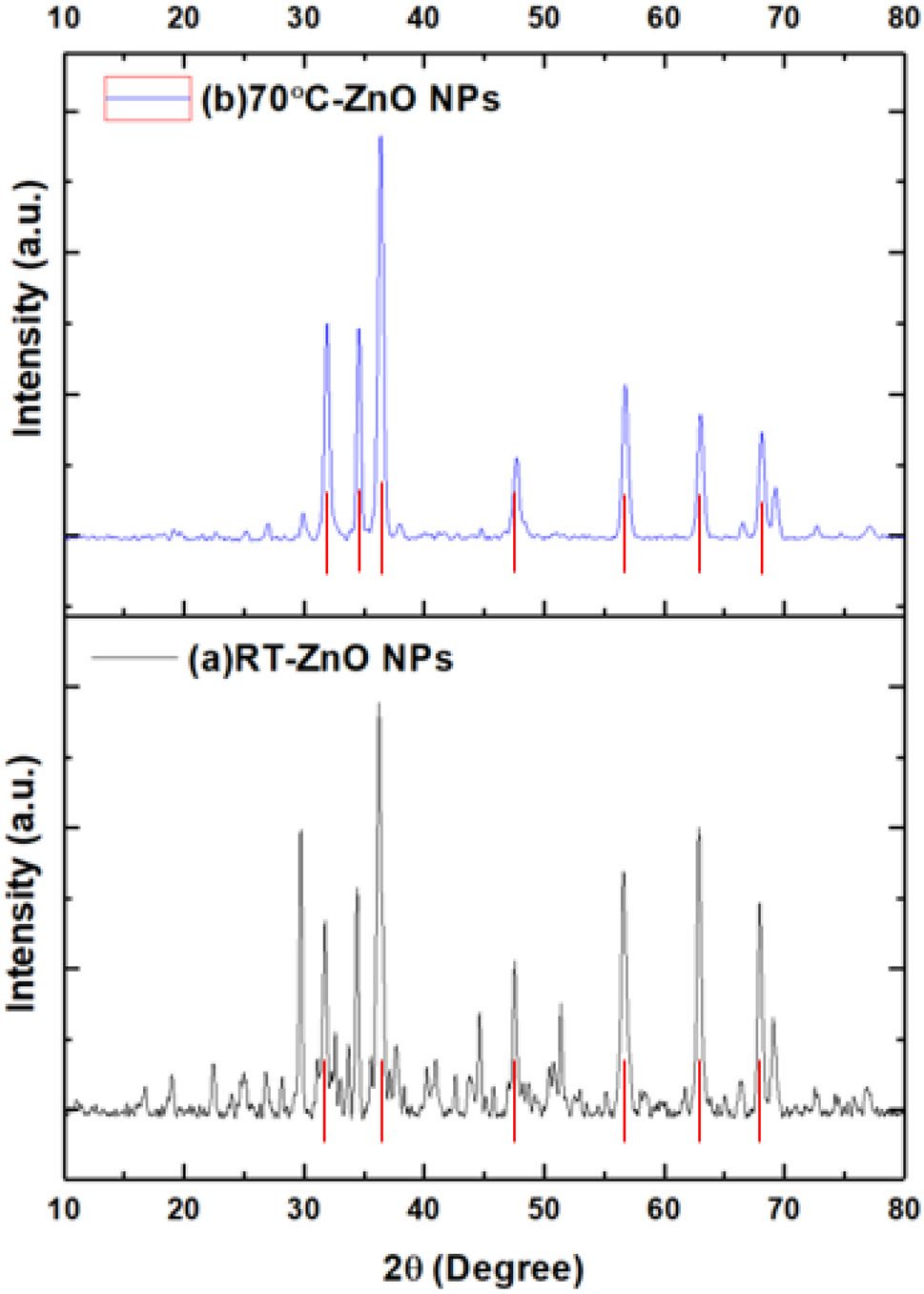
XRD pattern of the phyto-synthesized ZnO NPs, (**a**) RT, (**b**) 70 °C.

The sharp peaks exhibited by the prepared ZnO NPs confirmed their crystalline nature (Fig. 5). Diffraction peaks of rod-shaped ZnO NPs were at ~ 31.8°, 34.1°, 36.4°, 47.8°, 56.8°, 62.9° and 68.2°. The peaks of the hexagonal ZnO NPs were located at ~ 32.01°, 34.3°, 36.6°, 47.9°, 57.01°, 63.07°, and 68.4°. The peaks were compatible with JCPDS Card (2300112) for rod-shaped ZnO NPs and (1011258) for hexagonal NPs. The ZnO NPs attained a space group P 63 mc (no. 186), which was also established by other previous studies^29,59^.

The detected slight XRD peaks could be attributed to the crystallization of the detected phytochemicals (capping and stabilization of ZnO NPs) as was cited in the literature^59^. Using the Debye–Scherrer’s equation^60^, the average crystalline sizes of rod-shaped and hexagonal ZnO NPs were ~ 12 nm and ~ 11 nm, respectively. The size of NPs differs by using XRD and TEM as XRD provided data about the grain size which might be related to the polycrystalline of large particles resulting from the fusion of smaller grains^61,62^.

#### Thermogravimetric analysis (TGA)

**Figure 6:**
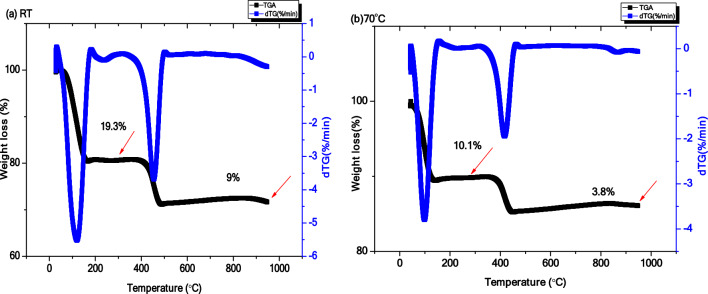
TGA analysis of the phyto-synthesized ZnO NPs (**a**) RT, (**b**) 70 °C.

As shown in the thermogravimetric analysis (Fig. 6), it demonstrates the weight loss (%) of ZnO NPs samples in response to temperature increases. The produced ZnO NPs proved remarkable thermal stability. The mass loss profile was steady up to 28.3 and 13.9% of the actual mass for nano-rods and the hexagonal NPs, respectively. This loss might be due to the elimination of moisture and carbonaceous phytochemicals in the prepared samples of ZnO NPs^22,63^.

The TGA analysis revealed two distinctive phases of weight loss. The TGA pattern of Rod ZnO NPs-RT (Fig. 6a) revealed a weight loss of 19.3% up to 296°C at the first phase and a slight weight loss of 9% from 296 to 947 °C at the second phase. A slightly higher thermal stability pattern was obtained by hexagonal ZnO NPs-70 °C (Fig. 6b) as it showed minor weight loss of 10.1% up to 286 °C at the first phase and 3.8% from 286 to 947 °C at the second phase. The initial weight loss might be due to moisture elimination and the second weight loss might be related to the decomposition of Zn (OH)_2_ to ZnO NPs^64–66^.

### Anticancer activity and cytotoxicity

**Figure 7:**
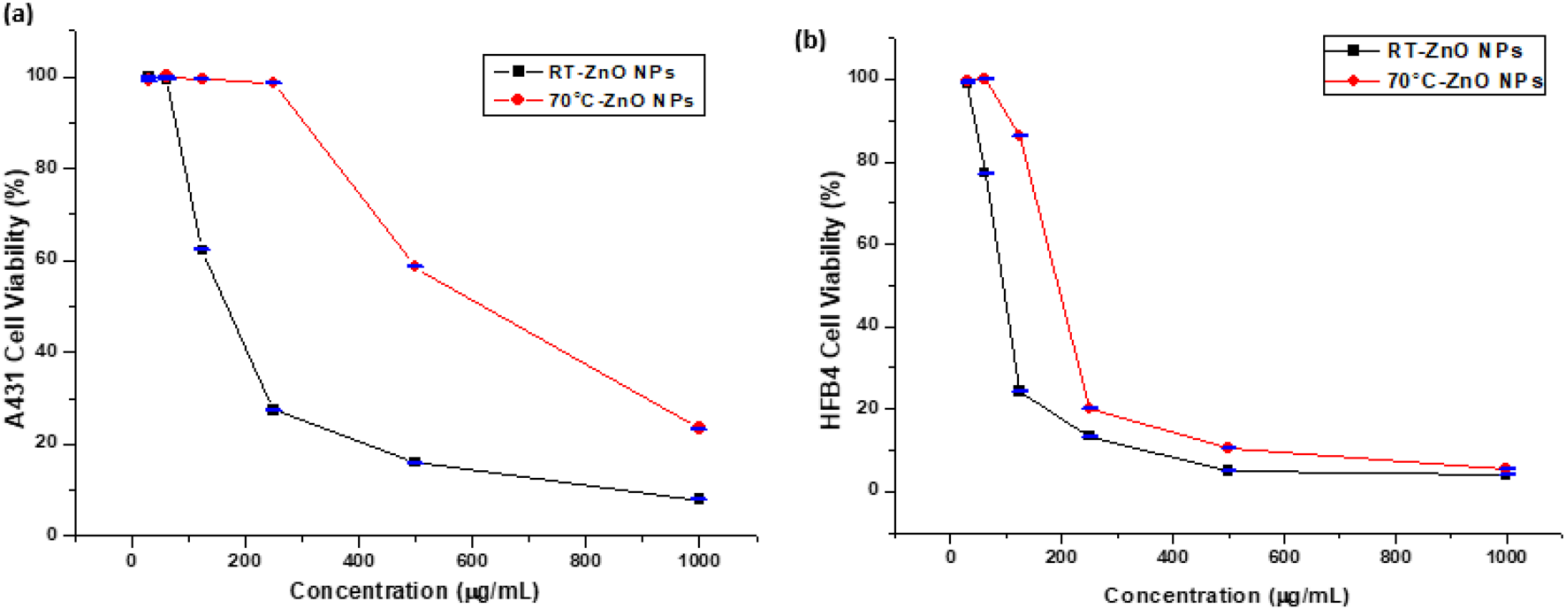
Cytotoxicity of the phyto-synthesized ZnO NPs against (**a**) A-431 cancer cell lines, (**b**) HFB4 normal cell lines. Data are expressed as mean ± SD, (n = 3).

The results showed that the rod-shaped ZnO NPs at RT had superior cytotoxicity toward cancer cells than hexagonal ZnO NPs at 70 °C. The IC50 of the phyto-synthesized rod-shaped ZnO NPs and hexagonal NPs was 93.88 ± 1 μg mL^−1^ and 187.15 ± 0.86 μg mL^−1^ respectively, against A-431 cancerous cell lines. This could be attributed to the small diameter and surface morphology of the obtained NPs^67^. Similar studies confirmed the cytotoxicity of ZnO NPs against A-431 cell lines (Fig. 7a). For instance, Chelladurai et al.^68^ proved that the IC_50_ of ZnO NPs was 125.3 μg mL^−1^ using *Alpinia calcarata* rhizome extract. In addition, Naiel et al.^36^ demonstrated that the IC_50_ for *Limonium pruinosum* aqueous extract was 409.7 μg mL^−1^ ZnO NPs. This finding confirms that ZnO NPs derived from *L. monopetalum* aqueous extract had higher cytotoxicity than previously reported.

The MTT assay results that were obtained after the cell lines were exposed to the produced nanoparticles, revealed that the ZnO NPs had a significant impact on the viability of the tumor cells. Using a concentration of 125 g mL^−1^ of rod-shaped ZnO NPs, the viability of the malignant cells significantly decreased to 24.3%. At a concentration of 250 g mL^−1^, the hexagonal ZnO NPs significantly decreased the viability of tumorous cells to 20.08%. Ali et al.^29^ indicated that the synthesized ZnO nano-rods from *Leea asiatica* leaf extract could be used as a substitute anticancer medication. Sadhukhan et al.^69^ revealed the functionality of ZnO nano-rods for bio-imaging and drug delivery usages. The prospective anticancer effect of ZnO NPs may be connected to the release of zinc ions, high oxidative stress, and finally cell apoptosis through DNA impairment and protein denaturation^70^.

Both obtained ZnO NPs showed higher cytotoxicity for cancer cells compared to normal cells. The IC_50_ of the phyto-synthesized rod-shaped ZnO NPs and near hexagonal NPs was 179.61 ± 3.63 μg mL^−1^ and 684.42 ± 19.1 μg mL^−1^ respectively against normal HFB4 cell lines (Fig. 7b). These findings confirm the reliability, and selectivity of phyto-synthesized ZnO NPs using *L. monopetalum* aqueous extract, which is in accordance with the findings by Babayevska et al.^13^, which proved that ZnO NPs and nano-rods were more destructive to cancerous cells than to the normal ones at the similar concentration.

The enhanced permeability and retention (EPR) effect may be related to the selectivity of ZnO NPs. They may increase the amount of zinc in cells, which may lead to protein disequilibrium and a rise in oxidative stress^11,71^.

**Figure 8:**
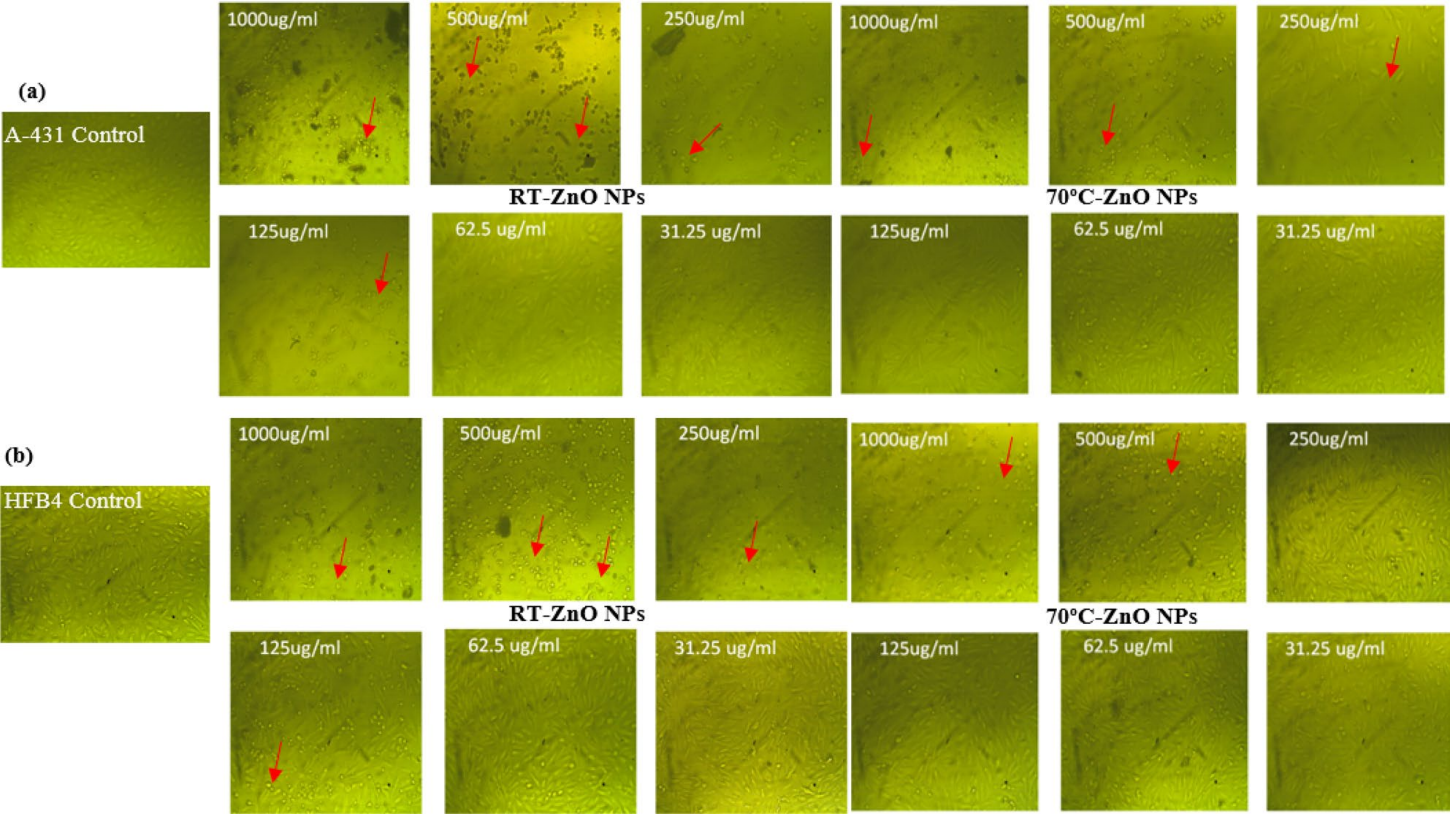
Morphological changes of (**a**) A-431 cancer cell lines and (**b**) HFB4 normal cell lines at different ZnO NPs concentrations.

Morphological changes in cancerous cells (A-431) and normal cells (HFB4) were also investigated as shown in Fig. 8a,b. These figures shows that at high concentrations of ZnO NPs, the cells became rounded and shrunk indicating the cytotoxic effect of those concentrations. It was detected that high concentrations can raise ROS levels, leading to DNA fragmentation, cell shrinkage and apoptosis^67,72^. The produced ZnO NPs appear to be more toxic to A-431 cell lines, confirming the biocompatibility of ZnO NPs.

### Antioxidant activity

**Figure 9:**
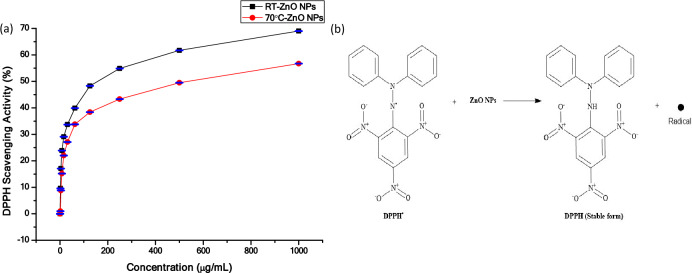
(**a**) The antioxidant activity of phytosynthesized ZnO NPs at RT and 70 °C. (**b**) The possible antioxidant mechanism of phyto-synthesized ZnO NPs. Data are expressed as mean ± SD (n = 3).

The antioxidant effect estimated for the two phyto-synthesized forms of ZnO NPs using DPPH assay revealed that both showed a concentration dependent free radical scavenging activity as was indicated in previous studies^73^. The rod-shaped ZnO NPs exhibited higher DPPH scavenging activity than hexagonal NPs (Fig. 9a). IC_50_ was 148.43 and 475.7 µg mL^−1^ for the phyto-synthesized rod-shaped ZnO NPs at RT and hexagonal ZnO NPs at 70 °C respectively. The small diameter of rod-shaped ZnO NPs relative to that of the hexagonal NPs might be the cause of their higher antioxidant capability^74^. In contrast to the current study, Saleemi et al.^75^ found low antioxidant activity for ZnO NPs with an IC_50_ > 500 µg mL^−1^. The phytochemicals that were related to reducing and stabilizing ZnO NPs might be responsible for their scavenging capabilities as stated by Sivasankarapillai et al.^43^.

The resultant formation of the hydroxyl and hydrogen radicals, may be responsible for the potential antioxidant effect of the synthesized ZnO NPs (Fig. 9b) as reported in other literature^43,76–78^. The spectrophotometric assessments of antioxidants' ability to scavenge DPPH radicals serve as the basis for this assay. Taking a hydrogen atom from the antioxidants reduces the solitary electron of the nitrogen atom in DPPH to hydrazine^79^.

### Antimicrobial activity

The merit of sustainable nanofabrication is to promote the use of nanomaterials as disinfectants against pathogenic microorganisms in water and wastewater. Water conventional methods for disinfection applying chlorination and ozonation create toxic and harmful byproducts^80^. While the sustainable nanomaterials could minimize the generated byproducts during the disinfection and corrosive suppression^81^.

Food-borne diseases have also exerted pressure on health and subsequent economic sectors. These pathogens can be transmitted via contaminated irrigation water, soil, and manure to various crops and eventually to humans^82^. Fungal pathogens, for instance, *Candida* is a significant cause of invasive infections, linked to high morbidity, mortality rates and prolonged hospitalization with high costs. Some fungal pathogens can also produce secondary byproducts that contribute to microbial corrosion in water distribution systems. *Candida* can exist in aquatic ecosystems contaminated with human or animal feces and also in potable water in hospitals^83–85^. *Aspergillus flavus* can grow in different kinds of food and produce toxic aflatoxins^86^. One of the main complications of fungal and bacterial species is the deleterious impacts on public health (see Table S1).

The results (Table 3) demonstrated that the ZnO NPs produced through phytosynthesis means exhibited a wide range of antimicrobial efficacy. When utilizing hexagonal ZnO NPs at 70 °C in comparison to the common antibiotic Gentamycin, the broadest inhibition zone was seen against Gram-negative bacteria *Pseudomonas aeruginosa* (ATCC 90274) at 30 mm and Gram-positive Bacteria *Bacillus Subtilis* (ATCC 6633) at 29 mm. These findings are in line with those of earlier research investigations^87–89^.

**Table 3:** Zones of inhibition in mm of different phytosynthesized ZnO-NPs against pathogenic bacteria and fungi.

Pathogenic microorganism	Rod ZnO NPs-RT	Hexagonal ZnO NPs-70 °C	Control*
*B. Subtilis* (ATCC 6633)	26	29	25
*P. aeruginosa* (ATCC 90274)	26	30	24
*S. aureus* (ATCC 6538)	23	26	23
*E. coli* (ATCC 8739)	20	21	19
*C. albicans* (ATCC 10,221)	21	26	23
*A. flavus*	NA**	NA**	17

*Control: Gentamycin for bacteria and Fluconazole for fungi.

**NA: No Activity.

Hexagonal ZnO NPs revealed slightly higher antibacterial and antifungal efficacy against all tested microorganisms except *Aspergillus flavus*. The efficacy of the antibacterial activity of ZnO NPs may be ascribed to their shape, morphology, and surface-to-volume ratio^67,90^. According to previous literature, the current findings proved higher inhibition zones applying the phyto-synthesized ZnO NPs as concluded in (Table 4).

**Table 4:** Antimicrobial activity of phyto-synthesized ZnO NPs produced in this work compared to the previously reported literature.

Phyto-synthesized ZnO NPs	Pathogenic microorganism	Inhibition zone (mm)	References
*Beta vulgaris-ZnO NPs*, *NPs*	*Escherichia coli*	10	^91^
	*Staphylococcus aureus*	0	
	*Candida albicans*	0	
*Cinnamomum tamala-ZnO*	*Escherichia coli*	8	
	*Staphylococcus aureus*	8	
	*Candida albicans*	0	
*Brassica oleracea var. italic*	*Escherichia coli*	10	
	*Staphylococcus aureus*	8	
	*Candida albicans*	8	
*Acalypha fruticose* -ZnO NPs	*Staphylococcus aureus*	21	^26^
	*Bacillus Subtilis*	21	
	*Pseudomonas aeruginosa*	16	
	*Candida albicans*	18	
*Boerhavia diffusa linn* seeds-ZnO NPs	*Staphylococcus aureus*	16	^2^
Rutin-ZnO NPs	*Escherichia coli*	9.376	^75^
	*Staphylococcus aureus*	9.563	
*L. monopetalum-Rod ZnO NPs-*RT	*Bacillus Subtilis*	26	Current study
	*Staphylococcus aureus*	23	
	*Escherichia coli*	20	
	*Pseudomonas aeruginosa*	26	
	*Candida albicans*	21	
	*Aspergillus flavus*	0	
*L. monopetalum Hexagonal ZnO NPs-*70 °C	*Bacillus Subtilis*	29	Current study
	*Staphylococcus aureus*	26	
	*Escherichia coli*	21	
	*Pseudomonas aeruginosa*	30	
	*Candida albicans*	26	
	*Aspergillus flavus*	0	

According to these findings, the antimicrobial effect of the produced ZnO NPs might be attributed to the capability to bind to the microbes’ cell membrane and enhancing the formation of ROS leading to oxidative stress, DNA damage, and finally cell death^26,42,73,92^. Sportelli et al.^10^ also demonstrated that zinc oxide has outstanding antimicrobial action, particularly at the nanoscale. Additionally, the FT-IR and GC–MS results recognized the occurrence of capping and stabilizing phytochemicals such as monoterpenoid alcohols, esters, and fatty acid methyl esters. These bioactive constituents demonstrated good antibacterial and antifungal activity, as mentioned by Zhumakanova et al.^93^.

## Conclusion and recommendations

Our results represent an added value for one of the halophytic species that always has been over looked. Successfully, the aqueous extract of *Limoniastrum monopetalum* (L.) Boiss mediated the synthesis of two different shapes of ZnO NP for the first time.

The results also demonstrated that reaction temperature played a major role in influencing the morphology, size, and physio-chemical characteristics of the obtained NPs. These outcomes were affirmed through the TEM images that proved the materialization of rod-shaped ZnO NPs at ambient temperature whereas hexagonal ZnO NPs were formed at 70 °C. The potential phytochemicals accountable for the reduction of ZnO NPs include monoterpenoid alcohols, esters and fatty acid methyl esters were identified using GC–MS and FT-IR. The anticancer, antimicrobial, and antioxidant investigations revealed the superior efficacy of the rod-shaped ZnO NPs relative to the hexagonal ones. The IC_50_ of rod-ZnO NPs was 93.88 ± 1 µg mL^−1^ against A-431 cancerous cell lines compared to 187.15 ± 0.86 μg mL^−1^ for hexagonal NPs. According to the results of the cytotoxicity, the IC_50_ values for rod-shaped ZnO NPs and hexagonal NPs against normal HFB4 cell lines were 179.61 ± 3.63 g mL^−1^ and 684.42 ± 19. g mL^−1^, respectively. This underscores the high cytotoxicity of the phyto-synthesized ZnO NPs toward skin cancer cells relevant to the normal cells and their biocompatibility. They also exhibited dose-dependent antioxidant activities, particularly rod-shaped ones. This study also emphasizes the significant effect of ZnO nanoparticle shape on pharmacological activity. Moreover, the two produced ZnO NPs forms can provide a potentially efficient substitute as an effective antimicrobial agent to eradicate resistant bacterial and fungal pathogens which is a challenge in water and wastewater disinfection. In conclusion, these results highlight the prospect of using *L. monopetalum* for ecofriendly synthesis of ZnO NPs with diverse morphologies in numerous prospective biomedical and environmental applications. Accordingly, this study adds more support for the transformation to green chemistry approach, thereby reducing the harmful effects of high energy consumption, pressure and hazardous chemicals during production. It also calls for the conservation of a highly vulnerable habitat, salt marshes, for pronounced role of halophytes in CO_2_ sequestration and contribution for climate change mitigation and adaptation measures.

### Supplementary Information

Supplementary Information.

## Data Availability

All data generated or analyzed during this study are included in the published article (and its Supplementary Information file).
