# Supplementary material for: Sustainable fabrication of dimorphic plant derived ZnO nanoparticles and exploration of their biomedical and environmental potentialities
Source: Sci Rep. 2024 Jun 12;14:13459. doi: 10.1038/s41598-024-63459-0 (PMC11167042; doi:10.1038/s41598-024-63459-0)
Supplement: Supplementary file 1 — Supplementary Information. [file 41598_2024_63459_MOESM1_ESM.docx]

Supplementary Information

**Sustainable fabrication of dimorphic plant derived ZnO nanoparticles and exploration of their biomedical and environmental potentialities**

Bassant Naiel^1*^, Manal Fawzy^1,2,3^, Alaa El Din Mahmoud^1,2^, Marwa Waseem A. Halmy^1,3^

^1^Environmental Sciences Department, Faculty of Science, Alexandria University, 21511, Alexandria, Egypt.

^2^Green Technology Group, Faculty of Science, Alexandria University, 21511, Alexandria, Egypt.

^3^National Egyptian Biotechnology Experts Network, National Egyptian Academy for Scientific Research and Technology, Egypt.

*Corresponding author: Bassant Naiel; bassant_hassan22@yahoo.com

**Material and methods:**

**S1. ZnO NPs physicochemical characteristics**

A double-beam UV/VIS Spectrophotometer (T70/T80 series PG Instruments Ltd, U.K.) with a 200–800 nm scanning wavelength range was used for the preliminary confirmation of ZnO NPs formation.

The functional groups and potential phytochemicals accountable for the reduction of Zn (CH_3_COO)_2_·2H_2_O salt in the inspected plant extract used in the synthesize of ZnO NPs were identified via scanning the produced material in the 4000–400 cm^_1^ wavelength region by Nicolet iS50 Fourier Transform Infrared Spectrometer (Thermo Fischer Scientific, Japan). The sample was prepared by mixing a small quantity of the powdered ZnO NPs with a powdered potassium bromide KBr, pressed and measured.

The identification of the phytochemical compounds was performed based on Naiel et al., [1].

The two produced preparations of ZnO NPs were inspected using TEM JEOL, Japan (JSM-1400 PLUS) for a precise morphological characterization. The Energy Dispersive X-Ray Analysis (EDXA-APEX) package was used for the configuration of the elemental composition of the produced material. The crystallinity of the two phyto-synthesized ZnO NPs was examined by Bruker D8, USA. X-ray diffractometer with Cu-Kɑ radiation (λ=1.54060 A°), the 2Ө range from 5 to 100 degrees.

In addition, thermogravimetric investigation to check their thermal stabilities was performed by the Labsys evo Setaram, France, in which exposing the nanoparticles to thermal fluxes starting from ambient temperature reaching to 1000ºC at a rate of 10ºC/min under a nitrogen atmosphere.

**S2. Anticancer activity and cytotoxicity:**

A tissue culture plate of 96-wells was inoculated with 1 X 10^5^ cells/ml (100 µl/well), then incubated for 24 hours at 37°C. After incubation, 20µl of MTT solution (5mg mL^-1^ in PBS) was added and incubated again (37^º^C, 5% CO_2_) for 4 hours to allow for the metabolization of the MTT to form the purple formazan crystals. These crystals were dissolved in 200µl DMSO. Absorbance was assessed at 560 nm [2-4]. The experiment was implemented in triplicate. The ZnO NPs half-maximal inhibitory concentration (IC_50_) was assessed using dose-response curve. The morphological modifications in the cells were also examined.

**Results and discussion**

**Antimicrobial activity:**

**Table S1. Diseases caused by fungal and bacterial species.**

| **Microorganism** | **Diseases** | **References** |
| --- | --- | --- |
| *Bacillus Subtilis* (ATCC 6633) | Bacteremia, Endocarditis, Pneumonia, and Septicemia | [5] |
| *Staphylococcus aureus* (ATCC 6538) | Osteomyelitis, Skin infections, Pneumonia, Sepsis, Epidural Abscess, Meningitis, Urinary Tract Infection | [6] |
| *Escherichia coli* (ATCC 8739) | Acute diarrheas and gastroenteritis | [7] |
| *Pseudomonas aeruginosa* (ATCC 90274) | Bloodstream infections | [7] |
| *Candida albicans* (ATCC 10221) | Respiratory infection, cutaneous or  systemic infections | [8] |
| *Aspergillus flavus* | Bronchopulmonary infections, Orbital infections, Cutaneous aspergillosis, *Aspergillus* sinusitis and osteomyelitis | [9] |

**References**

1. Naiel, B., Fawzy, M., Halmy, M. W. A. & Mahmoud, A. E. D. Green synthesis of zinc oxide nanoparticles using Sea Lavender (Limonium pruinosum L. Chaz.) extract: characterization, evaluation of anti-skin cancer, antimicrobial and antioxidant potentials. *Sci Rep* **12**, (2022).
2. Slater, T. F., Sawyer, B. & Sträuli, U. Studies on succinate-tetrazolium reductase systems. III. Points of coupling of four different tetrazolium salts III. Points of coupling of four different tetrazolium salts. *BBA - Biochimica et Biophysica Acta* **77**, (1963).
3. Alley, M. C. *et al.* Feasibility of Drug Screening with Panels of Human Tumor Cell Lines Using a Microculture Tetrazolium Assay. *Cancer Res* **48**, (1988).
4. van de Loosdrecht, A. A., Beelen, R. H. J., Ossenkoppele, G. J., Broekhoven, M. G. & Langenhuijsen, M. M. A. C. A tetrazolium-based colorimetric MTT assay to quantitate human monocyte mediated cytotoxicity against leukemic cells from cell lines and patients with acute myeloid leukemia. *J Immunol Methods* **174**, (1994).
5. Baron, S. *Medical Microbiology. 4th edition*. *University of Texas Medical Branch at Galveston* (1996).  ​
6. Tong, S. Y. C., Davis, J. S., Eichenberger, E., Holland, T. L. & Fowler, V. G. Staphylococcus aureus infections: Epidemiology, pathophysiology, clinical manifestations, and management. *Clin Microbiol Rev* **28**, (2015).
7. Cabral, J. P. S. Water microbiology. Bacterial pathogens and water. *International Journal of Environmental Research and Public Health* vol. 7 Preprint at <https://doi.org/10.3390/ijerph7103657> (2010).
8. Caggiano, G. *et al.* Occurrence of fungi in the potable water of hospitals: A public health threat. *Pathogens* **9**, 1–12 (2020).  ​​
9. Krishnan, S., Manavathu, E. K. & Chandrasekar, P. H. Aspergillus flavus: An emerging non-fumigatus Aspergillus species of significance. *Mycoses* vol. 52 Preprint at <https://doi.org/10.1111/j.1439-0507.2008.01642.x> (2009).
